# Mechanism of karyopherin-β2 binding and nuclear import of ALS variants FUS(P525L) and FUS(R495X)

**DOI:** 10.1038/s41598-021-83196-y

**Published:** 2021-02-12

**Authors:** Abner Gonzalez, Taro Mannen, Tolga Çağatay, Ayano Fujiwara, Hiroyoshi Matsumura, Ashley B. Niesman, Chad A. Brautigam, Yuh Min Chook, Takuya Yoshizawa

**Affiliations:** 1 Department of Pharmacology, University of Texas Southwestern Medical Center, Dallas, TX USA; 2 College of Life Sciences, Ritsumeikan University, Shiga, Japan; 3 Department of Biophysics, University of Texas Southwestern Medical Center, Dallas, TX USA

**Keywords:** Biochemistry, Biophysics, Molecular biology, Structural biology, Diseases

## Abstract

Mutations in the RNA-binding protein FUS cause familial amyotropic lateral sclerosis (ALS). Several mutations that affect the proline-tyrosine nuclear localization signal (PY-NLS) of FUS cause severe juvenile ALS. FUS also undergoes liquid–liquid phase separation (LLPS) to accumulate in stress granules when cells are stressed. In unstressed cells, wild type FUS resides predominantly in the nucleus as it is imported by the importin Karyopherin-β2 (Kapβ2), which binds with high affinity to the C-terminal PY-NLS of FUS. Here, we analyze the interactions between two ALS-related variants FUS(P525L) and FUS(R495X) with importins, especially Kapβ2, since they are still partially localized to the nucleus despite their defective/missing PY-NLSs. The crystal structure of the Kapβ2·FUS(P525L)^PY-NLS^ complex shows the mutant peptide making fewer contacts at the mutation site, explaining decreased affinity for Kapβ2. Biochemical analysis revealed that the truncated FUS(R495X) protein, although missing the PY-NLS, can still bind Kapβ2 and suppresses LLPS. FUS(R495X) uses its C-terminal tandem arginine-glycine-glycine regions, RGG2 and RGG3, to bind the PY-NLS binding site of Kapβ2 for nuclear localization in cells when arginine methylation is inhibited. These findings suggest the importance of the C-terminal RGG regions in nuclear import and LLPS regulation of ALS variants of FUS that carry defective PY-NLSs.

## Introduction

**Figure 1:**
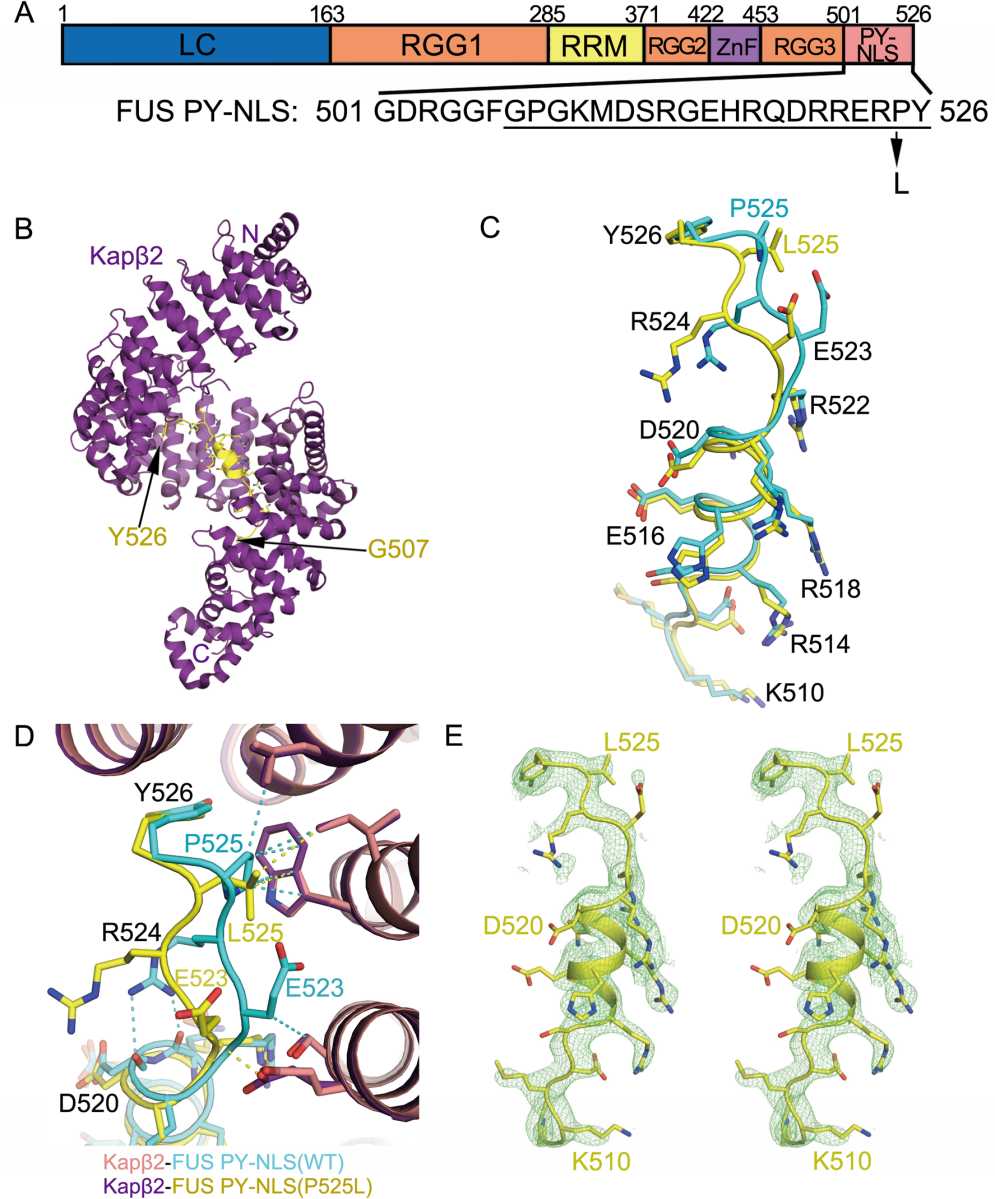
Structure of Kapβ2·FUS(P525L)^PY-NLS^ complex. (**A**) Sequence of FUS PY-NLS and the P525L mutation site. PY-NLS residues that are modeled in the Kapβ2·FUS(P525L)^PY-NLS^ crystal structure are underlined. (**B**) The overall structure of FUS(P525L)^PY-NLS^ (yellow) bound to Kapβ2 (purple). (**C**) Comparison of the Kapβ2-bound FUS(P525L)^PY-NLS^ (yellow) with WT FUS^PY-NLS^ (cyan). (**D**) Details of PY-NLS epitope-3 (FUS(P525L)^PY-NLS^ is in yellow; WT FUS^PY-NLS^ in cyan) interacting with Kapβ2 (purple and pink). Representative contacts ≤ 4.1 Å are shown as dashed lines. (**E**) Stereo view of the simulated annealing (SA) composite omit map 2Fo—Fc map contoured at 1.0σ overlaid onto the modeled mutant PY-NLS peptide.

Mutations in the Fused in Sarcoma (FUS) RNA-binding protein have been linked to the fatal neurodegenerative disease amyotrophic lateral sclerosis (ALS), as they cause approximately 5% of the cases for familial ALS and 1% of sporadic ALS^1^. Most disease-linked mutations of FUS are found in its C-terminal Proline-Tyrosine nuclear localization signal (PY-NLS), the RGG-rich regions (RGG) and the N-terminal prion-like or low-complexity region (LC) (domain organization of FUS is shown in Fig. 1A). The nuclear import receptor Karyopherin β2 (Kapβ2, also known as Transportin-1 or TNPO1) imports FUS into the nucleus, where it functions in DNA repair, transcriptional regulation, mRNA and miRNA processing, and in RNA shuttling and stabilization^2,3^. Wild type (WT) FUS localizes predominantly to the nucleus in healthy cells, but there is also a pool of FUS in the cytoplasm, which is especially evident in neuronal cells^4,5^. However, mutations related to the PY-NLS of FUS (FUS^PY-NLS^) cause the FUS variants to localize to different extents to the cytoplasm and cause different degrees of disease severity in patients. Two FUS variants, FUS(P525L) and FUS(R495X) cause the most severe disease^6–10^. ALS patients with the P525L mutations experience a severe type of juvenile ALS with an average disease onset age of 19.5 years, with onset as early as 11 years old^11^. The R495X nonsense mutation changes FUS residue Arg495 to a stop codon, and results in the deletion of its PY-NLS. ALS patients carrying the FUS(R495X) mutation experience early onset of symptoms at an average age of 35 years, with onset ages as early as 14 years^7,8^.

Kapβ2 imports WT FUS by binding tightly to the FUS^PY-NLS^ (residues 501–526), with a dissociation constant (K_D_) of ~ 50 nM. Crystal structures of Kapβ2 bound to the FUS^PY-NLS^ show that almost all FUS mutation sites found in ALS, including residue P525, make contacts with the importin^12^. Mutation of FUS Pro525 to leucine (P525L) decreased binding affinity for Kapβ2 by approximately tenfold, correlating well with the extent of FUS mislocalization to the cytoplasm^13,14^. Similar to the P525L mutation, the R495X nonsense mutation also result in accumulation of the FUS mutant protein in the cytoplasm^8,9,15^. Cytoplasmic localization of FUS(P525L) and FUS(R495X) further lead to the accumulation of the proteins into stress granules^8,13,14^.

Although the R495X mutation causes the nuclear protein FUS to accumulate in the cytoplasm, imaging studies of mammalian cells transfected with FUS(R495X) show that a fraction of the protein remains in the nucleus. Similarly, a small fraction FUS(P525L) localizes to the nucleus even though the protein is substantially mislocalized to the cytoplasm. It is unclear how the P525L missense mutation affects interactions of the PY-NLS with Kapβ2. It is also not known how FUS(R495X) is transported into the nucleus when it is missing the PY-NLS. Does it still bind Kapβ2 and be imported by this importin or does it bind other importins like the Importin-α/-β heterodimer (Impα/β), Importin-4 (Imp4), Importin-5 (Imp5), Importin-7 (Imp7), Importin-8 (Imp8), Importin-9 (Imp9), Importin-11 (Imp11) or Transportin-SR (Trn-SR or TNPO3) for nuclear import?

Here, we examine how FUS(P525L)^PY-NLS^ and FUS(R495X) interact with Kapβ2 and other importins that might support nuclear import. Although the FUS^PY-NLS^ peptide carrying the P525L mutation binds Kapβ2 weaker than the WT FUS^PY-NLS^, it still binds Kapβ2 with substantial affinity (K_D_ 180 nM), which allowed assembly of the Kapβ2·FUS(P525L)^PY-NLS^ complex for structure determination by X-ray crystallography. The crystal structure revealed that the P525L mutation resulted only in local changes such a shift of the main chain at position 525, which resulted in fewer contacts with Kapβ2. We also studied the interactions of FUS(R495X) and the slightly longer FUS(1–500) with Kapβ2 and other importins. Kapβ2 binds to both truncated FUS with lower affinities than for the FUS^PY-NLS^. FUS(1–500) binding to Kapβ2 is competitive with the FUS^PY-NLS^, suggesting that FUS(1–500) binds the PY-NLS binding-site of Kapβ2. FUS(1–500) also binds Impβ, but only in the absence of Impα. We mapped the interactions between Kapβ2 and FUS(1–500) to the C-terminal RGG2-ZnF-RGG3 segment of FUS. Affinity measurements of Kapβ2 binding to various FUS(1–500) domains/regions revealed that interactions with the RGG2 and RGG3 regions are key. In addition to binding Kapβ2, the C-terminal RGG2-ZnF-RGG3 fragment of FUS also binds Impβ, and binds weakly to Imp5, Imp8 and Imp9. In HeLa cells, FUS(R495X) localizes to the cytoplasm and the nucleus. Inhibition of arginine methylation increases its accumulation in the nucleus, supporting a role for its RGG regions. Inhibition by the M9M inhibitor further suggests that the RGG-mediate nuclear localization is mediated by Kapβ2.

## Results

### Structure of Kapβ2-FUS PY-NLS(P525L) complex

We solved the crystal structure of the FUS(P525L)^PY-NLS^ in complex with Kapβ2 (dissociation constant, K_D_ = 180 nM, Table 1) to 2.7 Å resolution, by molecular replacement (Fig. 1B–E). Crystallographic data and refinement statistics are shown in Supplementary Table 1. The complex crystallized in the same space group of *P*2_1_2_1_2 with similar crystallographic parameters as the Kapβ2·WT FUS^PY-NLS^^12^. Residues 507–526 of the bound FUS(P525L)^PY-NLS^ peptide were modeled. The FUS(P525L)^PY-NLS^-bound Kapβ2 is almost identical to WT FUS^PY-NLS^-bound Kapβ2 (root-mean square deviations or rmsd of 0.5 Å for 784 aligned Cα atoms; Fig. 1B,C). When Kapβ2 of the two structures are superimposed, it is obvious that the bound FUS(P525L)^PY-NLS^ peptide shows similar secondary structures as the bound WT FUS^PY-NLS^ and mostly binds Kapβ2 similarly (Fig. 1C). Epitope-1 (residues 508–511) and epitope-2 (residues 514–522) of FUS(P525L)^PY-NLS^ bind Kapβ2 almost exactly the same as WT PY-NLS (Fig. 1D). Structural differences are observed only in epitope-3 (residues 525 and 526) where the ALS mutation resides. The P525L mutation changes the structure of the PY-NLS main chain from residues 522–525, which diverged from the peptide main chain of the WT PY-NLS and moved slightly farther away from Kapβ2 (Fig. 1D,E). However, the Cα atoms of the last residues (Y526) are close again, resuming a position similar to that of Y526 in the WT PY-NLS. The distances between C_α_s of WT and P525L peptides are 2.3 Å, 3.0 Å, 1.7 Å and 1.1 Å for residues 523–526, respectively.

**Table 1:** ITC Measurements of Kap2 binding to FUS fragments.

FUS protein	Residues	K_D_ [2σ] ^a^
FUS^PY-NLS^	475–526	31 [17-50] nM
FUS^PY-NLS^ (P525L)	475–526	180 [130–240] nM
FUS(1–500)	1–500	160 [120–210] nM
FUS RGG2-ZnF-RGG3	371–500	170 [120–240] nM
FUS RGG2-ZnF	371–452	4 [2–11] μM
FUS RGG3	453–500	6 [3–10] μM
FUS LC-RGG1	1–370	35 [27–46] μM
FUS RRM	278–385	No Binding
FUS ZnF	415–460	No Binding

^a^The 95% confidence interval range for K_D_ determined by error-surface projection in the global analysis of the duplicates or triplicates of each experimental set.

In the WT PY-NLS, the P525 side chain makes hydrophobic interactions with the side chains of residues W460, I457 and L419 of Kapβ2. The L525 side chain of FUS(P525L)^PY-NLS^ makes fewer contacts with Kapβ2 W460 and I457, and is too far away to interact with the L419 side chain of Kapβ2 (Fig. 1D). There is no observed electron density for the E523 and R524 side chains of FUS(P525L)^PY-NLS^ even though these side chains have well-defined electron density in the Kapβ2-bound WT FUS^PY-NLS^ structure where E523 contacts the karyopherin and R524 participates in intra-peptide interactions (Fig. 1D,E)^12^. The latter interactions are likely important to stabilize the Kapβ2-bound NLS conformation, consistent with the multiple mutations at this position such as R524S/T/W found in ALS patients^16,17^. Loss of stable intra-peptide contacts by Arg524 in the FUS(P525L)^PY-NLS^ peptide may also contribute to decreased affinity for Kapβ2 as the mutant peptide is not preorganized into the Kapβ2-bound conformation. At the C-termini, the side chains of Y526 of both the WT and mutant PY-NLSs are in the same position, interacting with A381, D384 and L419 side chains of Kapβ2. In summary, the P525L mutation caused local structural changes that resulted in fewer contacts with Kapβ2 and in the loss of potentially stabilizing intra-peptide interactions, all contributing to a significant loss of affinity for the importin (Table 1).

### Impβ and Kapβ2 binds FUS(R495X) and inhibits its self-association

**Figure 2:**
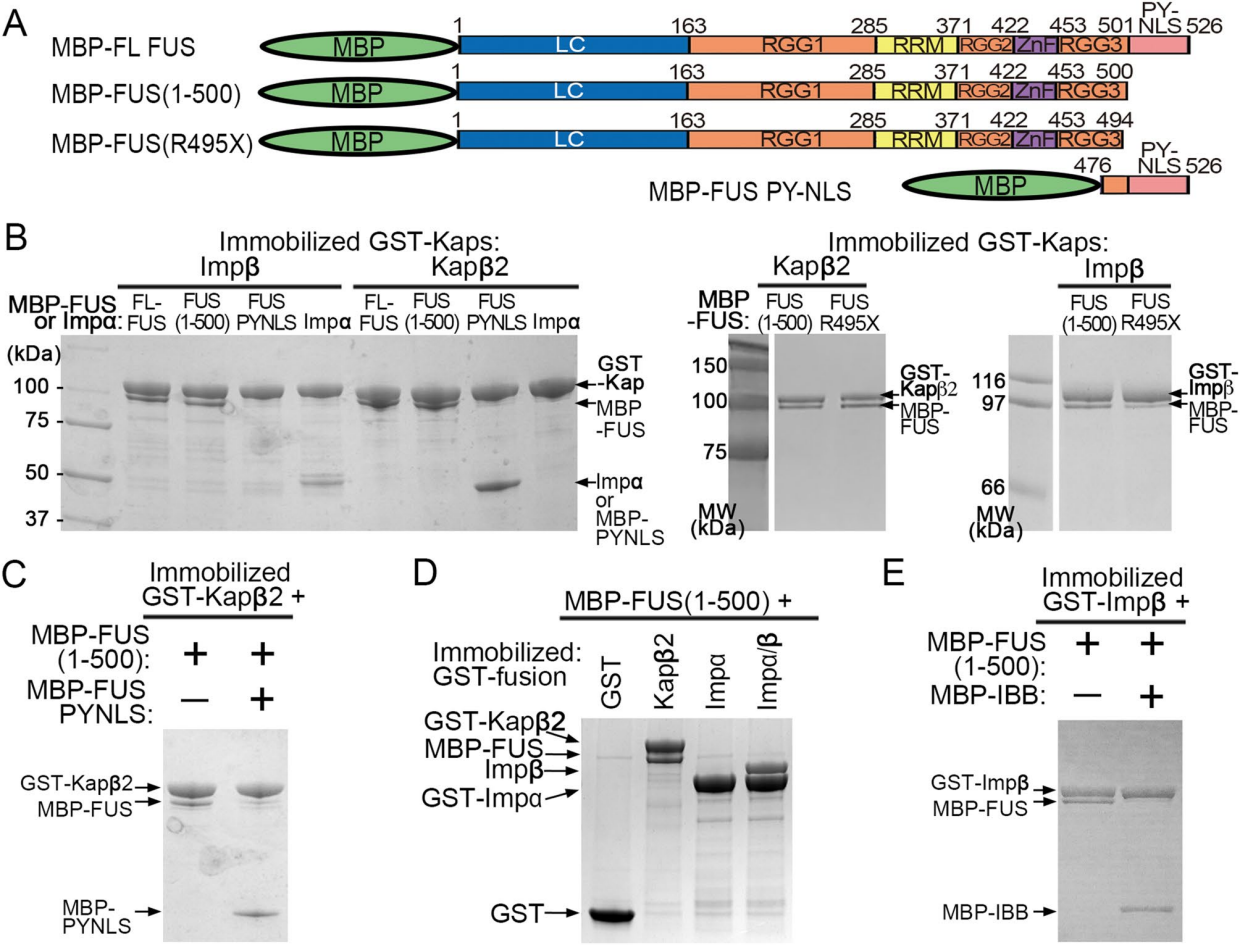
Interactions of FUS with Importins. (**A**) FUS constructs used in (**B**–**E**). (**B**) Pull-down binding assay showing interactions between MBP-FUS, MBP-FUS(1–500), MBP-FUS(R495X), MBP-FUS PY-NLS or Impα with GST-Impβ or GST-Kapβ2 immobilized on beads. Control experiments and unbound proteins in the pull-down experiments are shown in Supplementary Fig. 1A. (**C**) Pull-down binding assay of MBP-FUS(1–500) with immobilized GST-Kapβ2 in the presence of MBP-FUS PYNLS. (**D**) Pull-down binding assay to probe interactions between MBP-FUS(1–500) and GST-Kapβ2, -Impα or -Impα/β. (**E**) Pull-down binding assay of MBP-FUS(1–500) with immobilized GST-Impβ in the presence of MBP-IBB. Proteins in (**B–E**) are visualized by Coomassie-stained SDS-PAGE.

FUS(R495X) is localized to both the cytoplasm and the nucleus, but it is unclear how a fraction of the protein is transported into the nucleus when it is missing the PY-NLS, which is the key element for nuclear import of full-length (FL) FUS^8,13^. It is known that Kapβ2 binds tightly to the PY-NLS, and weakly and dynamically to all other regions of FUS^12,18^. It is not known if and how FUS(R495X) binds to Kapβ2 or other importins. We performed pull-down binding assays using GST-Impβ, GST-Impα and GST-Kapβ2 that are immobilized on beads and MBP-fusion proteins of FL-FUS, FUS(R495X) (residues 1–494), the slightly longer FUS(1–500) and the FUS^PY-NLS^ (residues 475–526) (Fig. 2A–E and Supplementary Fig. 1). As expected, Kapβ2 binds FL-FUS and FUS^PY-NLS^, but it also unexpectedly binds both FUS(R495X) and FUS(1–500) (Fig. 2B). Furthermore, although Impβ is not known to be a nuclear importer of FUS, it binds FL-FUS, FUS(R495X) and FUS(1–500) but not FUS^PY-NLS^ (Fig. 2B). Isothermal titration calorimetry (ITC) analysis shows that both FUS(R495X) (K_D_ = 330 nM) and FUS(1–500) (K_D_ = 161 nM) bind similarly to Kapβ2, but weaker than the FUS^PY-NLS^ (K_D_ = 31 nM) (Table 1, Supplementary Figs. S2 and S3). Kapβ2 binds FL FUS tightly through its PY-NLS, but can still bind the truncated protein with moderate affinity when the PY-NLS is not present. FUS(1–500) is only six amino acids longer than FUS(R495X) and clearly binds Kapβ2 and Impβ similarly. Many experiments below use FUS(1–500) as a proxy for FUS(R495X).

Figure 2C shows that Kapβ2 does not bind FUS(1–500) in the presence of FUS PY-NLS, suggesting that the binding site for FUS(1–500) on Kapβ2 likely overlaps with that for the PY-NLS. Analogously, although FUS(1–500) binds Impβ (Fig. 2B), it no longer does so in the presence of Impα and FUS(1–500) does not bind Impα (Fig. 2D). Therefore, it appears that FUS(1–500) binds directly to Impβ but cannot bind the Impα/β heterodimer. The Impβ-binding (IBB) fragment of Impα also inhibits Impβ interaction with FUS(1–500), suggesting that the binding site for FUS(1–500) on Impβ likely overlaps with that for the IBB (Fig. 2E).

**Figure 3:**
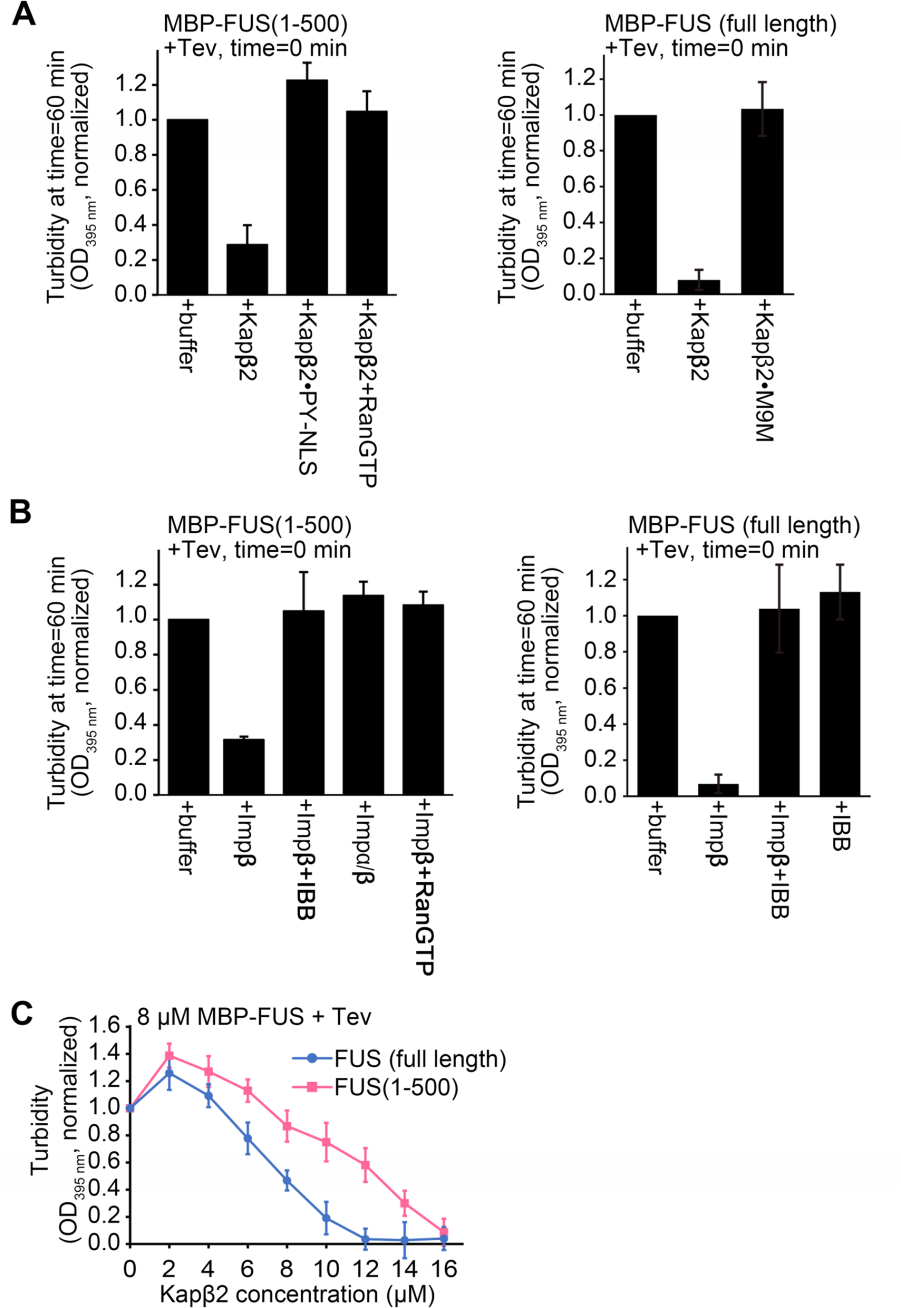
Turbidity assays of FUS in the presence of importins. (**A**) Left panel: turbidity assays with 8 μM MBP-FUS(1–500) in the presence of buffer, 8 µM Kapβ2, 8 µM Kapβ2·PYNLS and 8 µM Kapβ2 + 8 µM RanGTP. Right panel: turbidity assays of 8 µM MBP-FUS (full length) in the presence of buffer, 8 µM Kapβ2 or Kapβ2·M9M. (**B**) Left panel: turbidity assays of 8 μM MBP-FUS(1–500) in the presence of buffer, 8 µM Impβ, 8 µM Impβ + 8 µM IBB, 8 µM Impα/β, and 8 µM Impβ + 8 µM RanGTP. Right panel: turbidity assays of 8 µM MBP-FUS (full length) in the presence of buffer, 8 µM Impβ, 8 µM Impβ + 8 µM IBB and control of IBB alone. For turbidity assays In (**A**,**B**), importins and other proteins were added to the MBP-FUS proteins prior to addition of the Tev protease at time = 0 min, and OD_395 nm_ of the solutions measured 60 min after addition of the Tev protease. The experiments were performed at room temperature, OD_395 nm_ normalized to measurements of MBP-FUS proteins + buffer + Tev at time = 60 min, the mean of 3 replicate experiments, ± SD are shown. (**C**) Turbidity assays of 8 µM of three different MBP-FUS constructs in the presence of buffer or 2–16 µM Kapβ2 at 10 °C. Kapβ2 is added prior to Tev (added at time = 0 min) and OD_395 nm_ is recorded 60 min after Tev addition. OD_395 nm_ is normalized to measurements of respective MBP-FUS construct + buffer + Tev. Mean of 3 or 4 replicate experiments, ± SD is shown.

We had previously shown that FUS(1–500) and FL-FUS undergo liquid–liquid phase separation (LLPS) similarly^18^. Here, we examine how Kapβ2 and Impβ might control LLPS of FUS(1–500) and FL-FUS (Fig. 3A–C). Fusion of maltose binding protein (MBP) to the N-terminus of FUS prevents it from self-associating and undergoing LLPS, and purified MBP-FUS proteins appear unaggregated/monomeric by size exclusion chromatography^18^. We measured turbidity of the solution as MBP is cleaved away by the Tev protease from FUS(1–500) and FL-FUS, in the absence and presence of importins and/or other factors. (Fig. 3A, B). Kapβ2 inhibits both LLPS of FUS(1–500) and FL-FUS, consistent with interactions between Kapβ2 and the FUS constructs (Fig. 3A). This inhibition of LLPS is abolished in the presence of exogenous PY-NLS or M9M peptide inhibitor. The effect of exogenous PY-NLS is consistent with the binding results in Fig. 2C that suggest FUS(1–500) binding to the PY-NLS-binding site of Kapβ2. Impβ inhibits LLPS of both FUS(1–500) and FL-FUS (Fig. 3B), consistent with Impβ binding to FUS independently of its PY-NLS (Fig. 2B). This inhibition of LLPS is abolished in the presence of IBB or Impα (Fig. 3B), consistent with the binding results in Fig. 2D,E that suggests FUS(1–500) and FL-FUS binding to the IBB-binding site of Impβ.

To understand the FUS LLPS inhibition activity of Kapβ2 in more detail, we performed turbidity experiments for FL-FUS and FUS(1–500) with different concentrations of Kapβ2, at 10 °C. The lower temperature increased LLPS of the FUS proteins, allowing effects of the FUS constructs to be better distinguished. Kapβ2 inhibits LLPS of both FL-FUS and FUS(1–500) in a concentration-dependent manner (Fig. 3C). At any given concentration of Kapβ2, the importin inhibits LLPS of FUS(1–500) less than it does for FL-FUS, corresponding to the lower affinity for the former (Table 1).

### Importins binds the RGG2 and RGG3 regions of C-terminally truncated FUS

**Figure 4:**
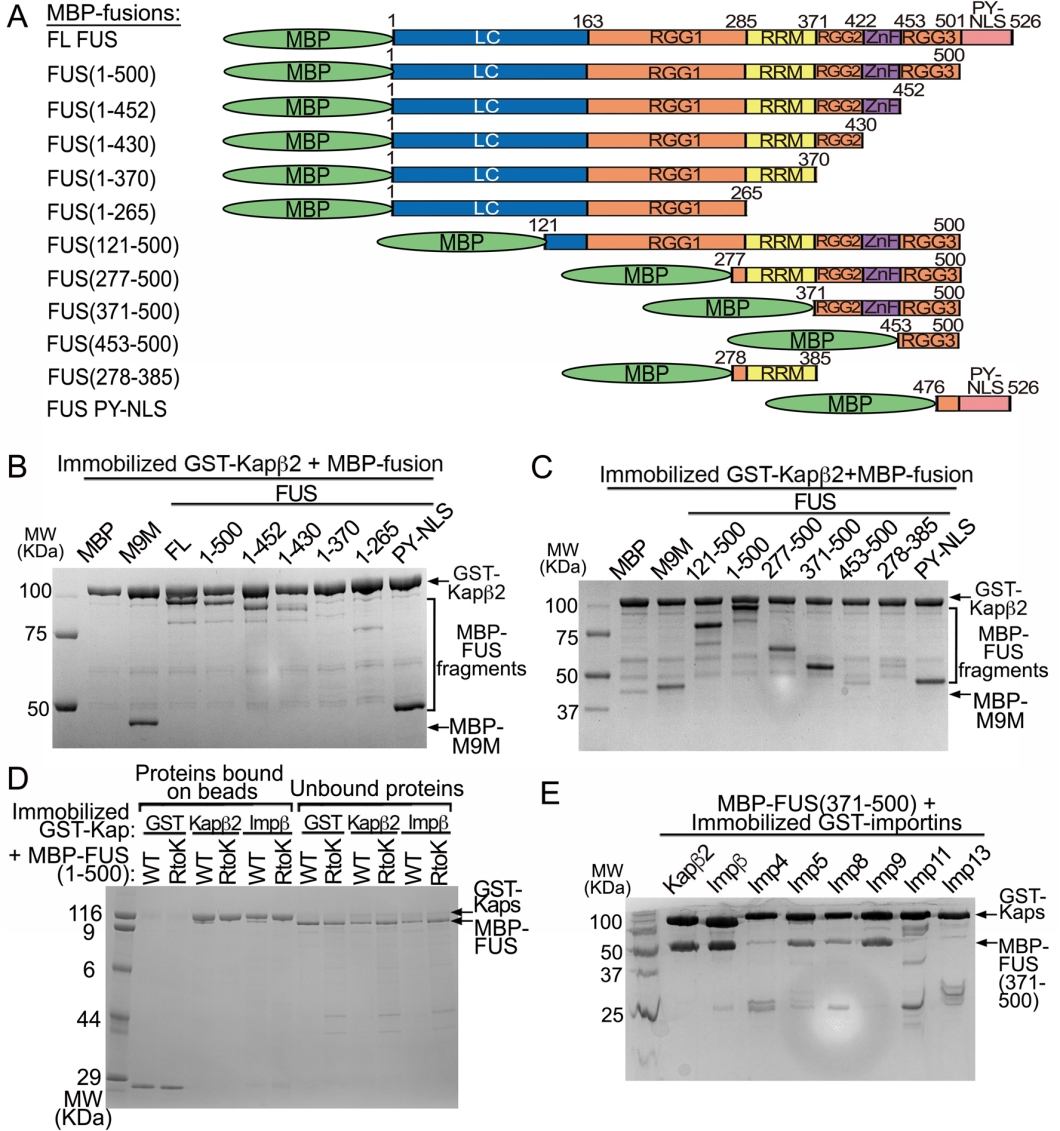
Interactions of Kapβ2 with various FUS fragments. (**A**) FUS constructs used in (**B**,**C**). (**B**,**C**) Pull-down binding assay showing the interactions of immobilized GST-Kapβ2 with various MBP, MBP-M9M and MBP-FUS constructs of various lengths. The MBP-FUS constructs in (**B**) have systematic deletions of regions from the C-terminus. Fragments containing residues 1–500 is missing the PY-NLS, 1–452 is missing RGG3-PYNLS, 1–430 is missing ZnF-RGG3-PYNLS, 1–370 is missing RGG2-ZnF-RGG3-PYNLS and 1–265 contains only LC-RGG1. All but one MBP-FUS constructs in (**C**) have no PY-NLS and systematic deletions of regions from the N-terminus. Fragments containing residues 121–500 is missing the LC and PY-NLS, 277–500 is missing LC-RGG1 and PY-NLS, 371–500 is missing LC-RGG1-RRM and PYNLS, 453–500 contains only RGG3 and 278–385 contains only the RRM**.** (**D**) Pull-down binding assay showing immobilized GST-Kapβ2 and GST-Impβ binds MBP-FUS(1–500) but not if all arginine residues in RGG2 and RGG3 were substituted by lysine (RtoK). (**E**) Pull-down binding assay of various immobilized GST-Importins with MBP-FUS 371–500. Proteins in (**B–E**) are visualized by Coomassie-stained SDS-PAGE.

To determine which region(s) in FUS(1–500) interacts with Kapβ2, we first performed pull-down binding assays of immobilized GST-Kapβ2 binding to multiple MBP-FUS constructs with systematic truncations from both termini (Fig. 4A–C). Proteins bound to beads are shown in Fig. 4B,C, and unbound proteins are shown in Supplementary Fig. 4A,B. We used MBP and MBP-M9M as negative and positive controls, respectively. Figure 4B and Supplementary Fig. 4A show binding assays for a series of FUS proteins truncated from the C-terminus. As expected, FL-FUS binds well to GST-Kapβ2. FUS(1–500), which is missing the PY-NLS, also binds well (Fig. 4A). Both FUS(1–452) and FUS(1–430), proteins further truncated to remove RGG3 or ZnF-RGG3, also bind GST-Kapβ2. However, when the RGG2 is removed, as in FUS(1–370) and FUS(1–265), little to no binding to GST-Kapβ2 is observed. Lastly, FUS(475–526) or the FUS^PY-NLS^, another positive control for the assay, binds well to GST-Kapβ2.

Figure 4C and Supplementary Fig. 4B show binding assays for several FUS proteins without the PY-NLS and are also truncated from the N-terminus. Removing the LC region in FUS(121–500) shows no discernible difference compared to FUS(1–500), in binding GST-Kapβ2. Further removal of RGG1 in FUS(277–500) also preserves GST-Kapβ2 binding. Removal of RRM in FUS(371–500) does not seem to affect binding of GST- Kapβ2, consistent with the no binding observed for the RRM alone construct (FUS(278–385)). However, further removal of RRG2-ZnF (FUS(453–500)) abolished binding to GST-Kapβ2. Altogether, results of the binding assays shown in Fig. 4B,C suggest that the C-terminal fragment of FUS that contains RGG2 and RGG3 are important for binding Kapβ2.

We performed ITC to measure equilibrium dissociation constants (K_D_s) of Kapβ2 binding to various domains and regions of C-terminally truncated FUS (Table 1 and Supplementary Fig. 2). For these ITC experiments, we controlled the quality of different Kapβ2 preparations by using only ones that show tight binding to MBP-hnRNP A1 PY-NLS (K_D_ ~ 50 nM)^19^. We also used size exclusion chromatography to ensure that all MBP-FUS proteins used are not aggregated. ITC experiments were performed with Kapβ2 in the cell and MBP-FUS in the syringe, except for MBP- FUS(R495X), MBP-FUS(1–500) and MBP-FUS(1–370) where the experimental setups were reversed. All ITC experiments were performed in either on triplicate or duplicate (Table 1 and Supplementary Fig. 2). As expected, MBP-FUS^PY-NLS^ (residues 475–526) binds Kapβ2 tightly (K_D_ 31 [17–50] nM), similar to previously reported affinities^12,18^. MBP-FUS(371–500), composed of RGG2-ZnF-RGG3, also binds Kapβ2 (K_D_ 170 [120–240] nM) with the same affinity as MBP-FUS(1–500) (K_D_ 160 [120–210] nM), which is approximately sixfold weaker than Kapβ2 binding to MBP-FUS^PY-NLS^. Constructs smaller than MBP-FUS(371–500) bound Kapβ2 significantly weaker: K_D_s of 4 μM and 6 μM for RGG2-ZnF (residues 371–452) and RGG3 alone (residues 453–500), respectively. Isotherms for the individual folded domains, RRM (residues 278–385) and ZnF (residues 415–460) suggest that they do not interact with Kapβ2 (Table 1 and Supplementary Fig. 2). The LC-RGG1 fragment (residues 1–370) binds Kapβ2 very weakly, with an apparent K_D_ of 35 μM. Altogether the ITC results suggest that RGG2 and RGG3 regions of FUS(1–500) and FUS(R495X) are key for binding Kapβ2.

In a previous study, we had shown that high affinity interactions of the PY-NLS in FL FUS anchors the protein to Kapβ2 and allows weak transient interactions involving the FUS N-terminal low complexity and C-terminal RGG regions that prevent FUS LLPS and aggregation^18^. Arginine residues in RGG2 and RGG3 play important roles in the weak and transient chaperoning contacts with Kapβ2. Here, we show that C-terminally truncated FUS variants that are missing the PY-NLS use RGG2 and RGG3 to bind Kapβ2 with moderate affinity. We wondered if arginine residues in RGG2 and RGG3 are also important for truncated FUS to bind importins. We mutated all arginine residues in the FUS(1–500) and in FUS(371–494) to lysines to assess their importance in binding Kapβ2. Both the FUS(1–500/RtoK) and FUS(371–494/RtoK) mutants do not bind Kapβ2 or Impβ (Fig. 4D and Supplementary Fig. 4C). These results confirm the importance of the arginine side chains (rather than positive charges alone) in RGG2 and RGG3 for moderate binding of truncated FUS proteins that are missing their PY-NLSs to importins.

Since Kapβ2 binds similarly to both the RGG2-ZnF-RGG3 fragment (residues 371–500) and FUS(1–500), we also investigated binding of the former to several different importins (Fig. 4E and Supplementary Fig. 4D). Control experiments of interactions with RanGTP verified that the importins are folded and active, but do not bind control beads with immobilized GST (Supplementary Fig. 4E,F). MBP-FUS(371–500) binds selectively to a few importins. It shows the most binding to Kapβ2 and Impβ, and slightly less binding to Importin-5 (Imp5) and Importin-9 (Imp9). MBP-FUS(371–500) shows little to no binding to Importin-4 (Imp4), Importin-8 (Imp8), Importin-11 (Imp11) and Importin-13 (Imp13). These results suggest that other than Kapβ2, importins such as Impβ, Imp5 and Imp9 may also be able to transport FUS(R495X) into the nucleus through interactions with its RGG2 and RGG3 regions.

In summary, both the RGG2 and RGG3 regions of FUS are used by C-terminally truncated FUS to bind Kapβ2. In FUS proteins that are missing the PY-NLS, the RGG2-ZnF-RGG3 fragment is necessary and sufficient for Kapβ2-binding. Furthermore, the FUS RGG2-ZnF-RGG3 can bind not only Kapβ2, but several other importins.

### Localization of FUS(R495X) and FUS(371–500) in cells

**Figure 5:**
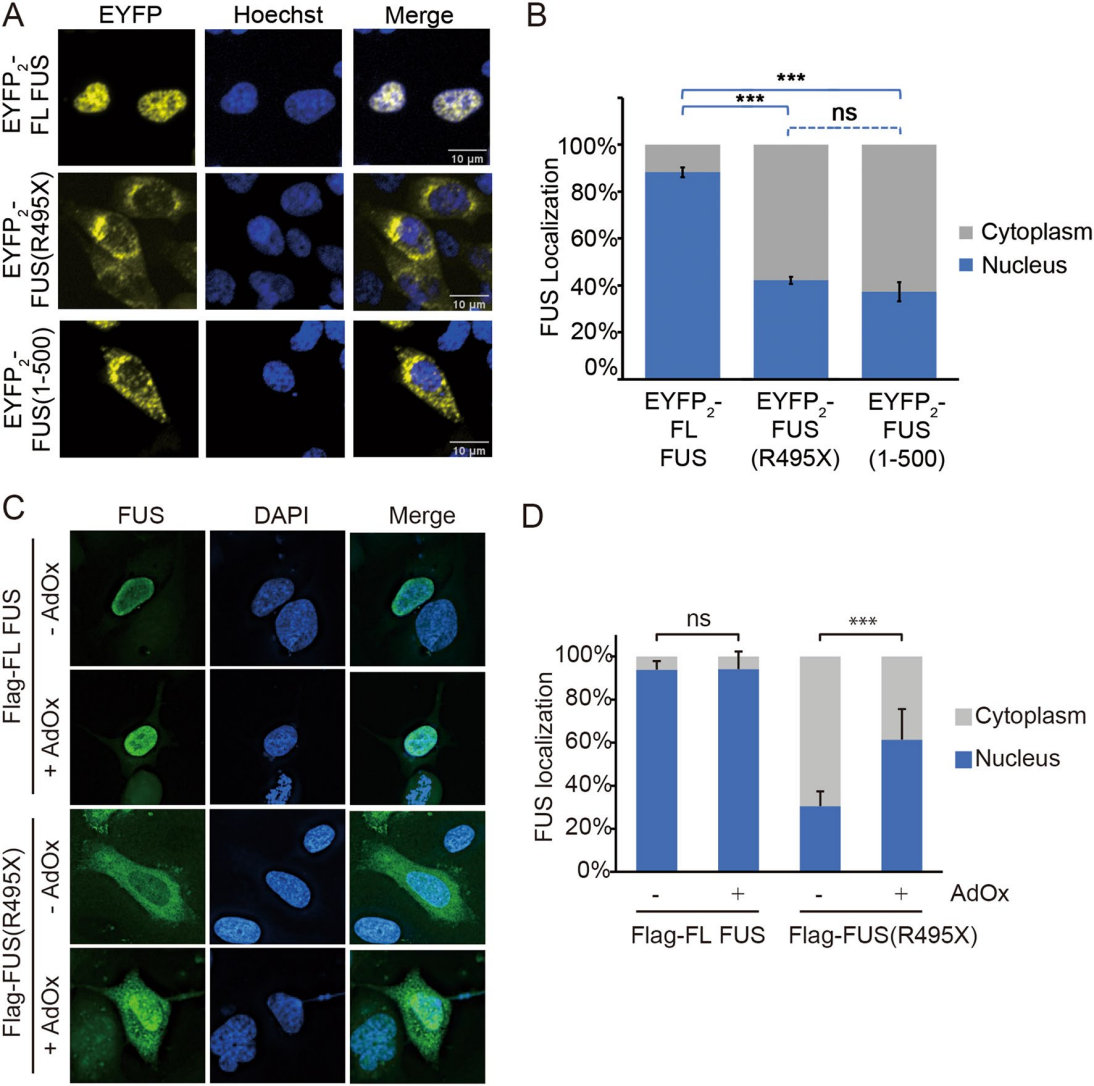
Localization of full-length FUS, FUS(R495X) and FUS(1–500) in HeLa cells. (**A**) Confocal microscopic images of live HeLa cells expressing EYFP_2_-FL FUS, EYFP_2_-FUS(R495X) or EYFP_2_-FUS(1–500). (**B**) Bar diagram of relative percentage of nuclear and cytoplasmic fluorescence intensity in cells, with the mean ± SEM, n = 10–14. Significant differences compared with the corresponding control samples are indicated ***p < 0.001. ns, not significant (Ordinary one-way ANOVA test). (**C**) Fluorescence microscopic images of fixed HeLa cells expressing Flag-FL FUS or -FUS(R495X), with or without methylation inhibitor AdOx (20 μM) treatment. FUS is visualized by immunofluorescence (Alexa 488 secondary antibody). (**D**) Bar diagram of relative percentage of nuclear and cytoplasmic fluorescence intensity of cells in (**C**). More than 30 cells were analyzed for each experiment. Error bars represent standard deviation. ***Indicates adjusted *P* values < 0.001.

**Figure 6:**
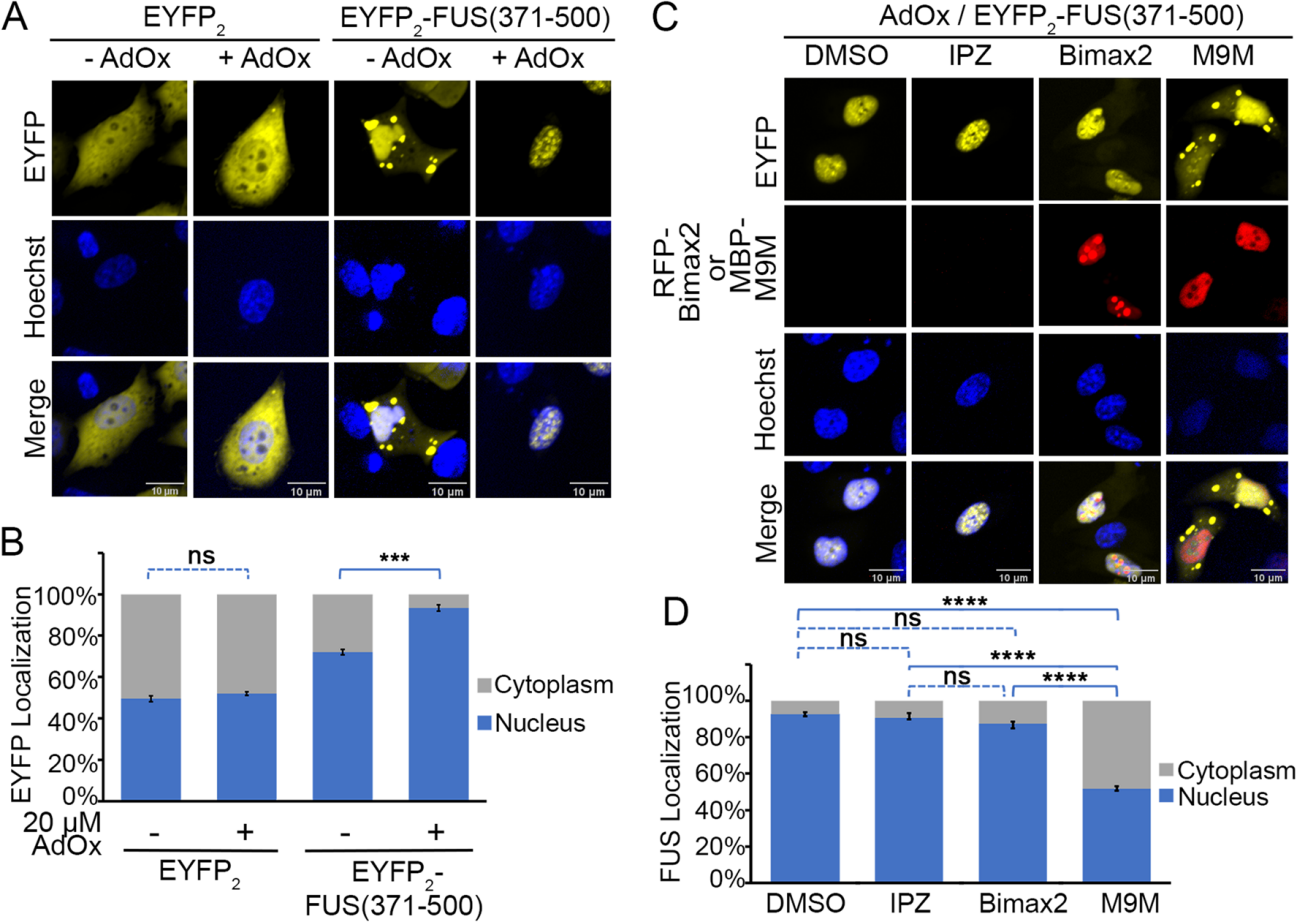
Localization of FUS(371–500) in the presence and absence of importin inhibitors. (**A**) Confocal microscopic images of live HeLa cells expressing EYFP_2_ or EYFP_2-_FUS(371–500), with or without methylation inhibitor AdOx (20 μM treatment. Hoechst 33,342 was used as nuclear counter stain. Scale bar = 10 μm. (**B**) Bar diagram of relative percentage of nuclear and cytoplasmic fluorescence intensity in cells is shown with the mean ± SEM, n = 10–14. (**C**) Localization of EYFP_2_-FUS(317–500) in the presence of importin β1 inhibitor (Importazole), importin α inhibitor (expressed Bimax2 peptide) and Kapβ2 inhibitor (expressed M9M peptide). The HeLa cells were first treated with AdOx. Expression of the peptide inhibitors were either monitored directly (RFP-BiMax2, second row) or by direct immunofluorescence (MBP-M9M via Alexa 565 s antibody, second row). Scale bar = 10 μm. (**D**) Bar diagram of relative percentage of nuclear (Nuc) and cytoplasmic (Cyto) fluorescence intensity in cells is shown with the mean ± SEM, n = 10–14. Ordinary one-way ANOVA test was performed for statistical analysis using GraphPad software. Significant differences compared with the corresponding control samples are indicated ****p < 0.001. ns, not significant.

Next, we examined the localization of C-terminally truncated FUS constructs in cells and the importance of RGG-importin interactions for nuclear import of these constructs. We expressed several FUS constructs that are tagged with tandem EYFP fluorescent proteins at their N-termini (EYFP_2_-FUS) in HeLa cells (Figs. 5A, B, 6A–D and Supplementary Figs. 5–7). EYFP_2_-FL-FUS is mostly localized to the nucleus whereas C-terminally truncated constructs EYFP_2_-FUS(R495X) and EYFP_2_-FUS(1–500) are localized almost evenly to both the nucleus and the cytoplasm (Fig. 5A,B). We also examined cellular localization of Flag-tagged FUS constructs.

Flag-tagged FL-FUS is localized mostly in nucleus but Flag-tagged FUS(R495X) is mostly in the cytoplasm (Fig. 5C,D).

Arginine residues in FUS RGG regions may be asymmetric demethylated in cells and methylation is known to decrease nuclear import^20–23^. We wondered if nuclear import of Flag- FUS(R495X) is significantly decreased because of arginine methylation. General methylation inhibition by the S-adenosylmethionine (SAM)-dependent methylation inhibitor adenosine-2′,3′-diadehyde (AdOx)^22,24^ increased nuclear localization of Flag-tagged FUS(R495X) (Fig. 5C,D), suggesting that the protein was indeed methylated in the absence of AdOx and that unmethylated arginine residues in its RGG regions are important for nuclear localization. It is not surprising that Flag-FL FUS is localized in nucleus regardless of AdOx treatment (Fig. 5C,D) since the PY-NLS that directs its nuclear import does not contain arginine methylation sites. Interestingly, the less cytoplasmic EYFP_2_-FUS(R495X) and EYFP_2_-FUS(1–500) and their unchanged localization when treated with AdOx suggest that there’s little methylation of these constructs in HeLa cells (Supplementary Fig. 5).

We also investigated cellular localization of smaller FUS fragments that contain only RGG2-ZnF-RGG3 (constructs FUS(371–500) and FUS(371–494)). FUS(371–500) binds Kapβ2 with the same affinity of FUS(1–500) (Table 1). Furthermore, these shorter fragments should not be ‘inhibited’ as FL FUS is, through interactions with the N-terminal LC region^25,26^. As predicted, EYFP_2_-FUS(371–500) and FUS(371–494) are mostly nuclear, but both constructs are also present in cytoplasmic puncta (Fig. 6 and Supplementary Fig. 6). AdOx treatment further increases nuclear location of both proteins and they no longer form cytoplasmic puncta (Fig. 6A,B and Supplementary Fig. 6A,B). These results suggest that the RGG regions of EYFP_2_-FUS(371–500) and EYFP_2_-FUS(371–494) are methylated in HeLa cells. Consistently, mutations of all arginine residues in EYFP_2_-FUS(371–494) to lysines (EYFP_2_-FUS(371–494/RtoK) render localization insensitive to AdOx (Supplementary Fig. 6A,B) and overexpression of arginine methyltransferase PRMT1 decreases nuclear localization of EYFP_2_-FUS(371–494) (Supplementary Fig. 7).

We co-expressed the M9M peptide inhibitor of Kapβ2 (as MBP-M9M) to test if Kapβ2 mediates nuclear import of EYFP_2_-FUS(371–500) or EYFP_2_-FUS(371–494)^27^. We also co-expressed a high-affinity peptide inhibitor of Impα (RFP-Bimax2) and a small molecule inhibitor of the Impβ (Importazole) to test involvement of Impα or Impβ (EYFP_2_-FUS(371–500) in Fig. 6C,D and EYFP_2_-FUS(371–494) in Supplementary Fig. 6C,D)^28,29^. The nuclear localization the FUS(371–500) was significantly decreased in cells expressing M9M. Note that nuclear FUS(371–500) levels are lower in M9M-expressing cells than untreated or minus AdOx cells.

FUS(371–500) also accumulates in cytoplasmic puncta, all suggesting involvement of Kapβ2 in nuclear localization of FUS(371–500) (Fig. 6C,D). Neither Importazole (IPZ) treatment nor Bimax2 expression had much effect on nuclear localization of FUS(371–500), suggesting minor or no involvement of Impα or Impβ in this process.

In summary, we showed that the RGG regions of C-terminally truncated FUS can mediate nuclear import, especially when they are not methylated. This RGG-mediated nuclear import is most likely mediated Kapβ2.

## Discussion

FUS(P525L) and FUS(R495X) are two FUS mutants that cause juvenile ALS. Since ALS is an aging disease, these FUS mutants are likely at least partially functional early in life, consistent with their partial nuclear localization observed in tissue culture cells. The preservation of FUS nuclear function in the FUS(P525L) is supported by the crystal structure of Kapβ2 bound to FUS(P525L)^PY-NLS^, which showed the mutant PY-NLS peptide binding in PY-NLS binding site of Kapβ2 in almost the same mode as the WT PY-NLS. Local shifts at position 525 of FUS results in fewer contacts with Kapβ2, explaining the decreased affinity and mislocalization to the cytoplasm^14,30^. Surprisingly, despite missing the PY-NLS, FUS(R495X) retains interactions with Kapβ2, which also inhibits its LLPS. This interaction is mediated by the RGG2-ZnF-RGG3 segment of FUS(R495X) binding to the PY-NLS binding site of Kapβ2. The same portion of FUS also binds Impβ, Imp5 and Imp9.

The ZnF domain of FUS does not interact with Kapβ2 and the interaction with FUS(R495X) appears to be mediated by both the RGG2 and RGG3 regions. The binding preference of RGG regions to importins is not surprising as the PY-NLS binding site of Kapβ2, the IBB-binding site of Impβ and multiple surfaces on Imp5 and Imp9 are acidic^19,31–33^. Multiple arginine residues in FUS RGG2 and RGG3 likely bind randomly in somewhat persistent manner to the same sites within the PY-NLS binding site of Kapβ2, resulting in a K_D_ of ~ 160 nM. We previously reported that when the PY-NLS of FL-FUS occupies Kapβ2′s PY-NLS binding site (K_D_ ~ 30 nM), the FUS RGG regions bind weakly and dynamically to site(s) outside of the PY-NLS binding site of Kapβ2 ^18^. Interestingly, Bourgeois et al. recently reported that the acidic loop of Kapβ2 interacts with RGG regions of cold-inducible RNA-binding protein, CIRBP^34^. Therefore, Kapβ2 and probably other importins may interact with RGG regions using many different modes.

Multivalent cation-π interactions between arginine residues in the RGG regions and tyrosine residues in the LC region are critical for LLPS and likely aggregation^25,35^. We showed that both Kapβ2 and Impβ can inhibit the LLPS of FUS(495X) by binding it using their PY-NLS and IBB binding sites, respectively. Importin interactions with the RGG regions are most likely important to prevent cation-π interactions with the LC and hence LLPS. Importin-RGG interactions beyond the FUS protein are likely important in cells as many proteins, especially RNA binding proteins, contain RGG/RG repeats, undergo LLPS and bind importins^36,37^. Importins may be involved in LLPS regulation of the variety RGG/RG repeats containing proteins.

FUS RGG regions, through hydrogen bonding and electrostatic interactions of their arginine residues, bind a variety of RNAs, including stem loop and guanine quadruplex structures. Each of the three RGG1, RGG2 and RGG3 regions contain 6–8 RG or RGG motifs, but they seem to have distinct RNA-binding specificity^38,39^. RGG1 binds 48-nucleotide G-quadruplex prD (DNMT) RNA. RGG2 and RGG3 also bind the prD G-quadruplex, but with weaker affinity than RGG1. RGG2 (with RRM and ZnF) binds the stem region of stem loop RNA^40^, it also binds other structures RNAs but generally with weaker affinities than RGG1 or RGG3, and it does not bind single strand RNAs. RGG3 binds the G-quadruplex TERRA RNA^41^. It appears that RGG1 and RGG3 may bind both structured and unstructured RNA elements while RGG2 thus far has been shown to bind only structured RNAs. We had previously reported Kapβ2 caused efficient release of the prD RNA (binds all RGGs; K_D_ ~ 0.7 μM) and the only partial release of the higher affinity the G-quadruplex TERRA (binds RGG3; K_D_ ~ 12 nM) from the respective MBP-FL FUS·RNA complexes, consistent with overlapping Kapβ2 (weak and transient interactions) and RNA binding sites in all three FUS RGG regions^18,38,42^. Since FUS(R495X) uses both RGG2 and RGG3 (but not RGG1) to bind persistently to Kapβ2, the FUS variant is unlikely to bind RNAs through RGG2 and RGG3 in the presence of Kapβ2. It is unclear at this time, if Kapβ2 binds weakly and transiently to the LC and RGG1 regions while it is anchored to the RGG2 and RGG3 of FUS(R495X), and if Kapβ2-binding would prevent RNA binding to the RGG1 of FUS(R495X).

ALS is an aging disease that is thought to be caused by multiple hits or deleterious events. The first of these hits could be mutations such as the R495X mutation in FUS, but the FUS(R495X) proteins are probably partially functional in the nucleus prior to disease onset. We showed here that FUS(R495X) can localize to the nucleus using its RGG2 and RGG3 regions, and nuclear import is likely mediated by Kapβ2 and possibly also other importins such as Imβ, Imp5 and Imp9. It is well established that methylation of arginine residues in RGG regions control nuclear localization of FUS^22,43^. We also found that inhibition of methylation increased nuclear accumulation of both FUS(R495X) and the RGG2-ZnF-RGG3 segment of FUS. It is possible that a later hit could result in increased methylation that would further deplete nuclear FUS. Similarly, additional defects in the nuclear transport machinery would have the same effects of depleting nuclear FUS, increasing accumulation of FUS in the cytoplasm leading to formation of FUS-containing disease inclusions and disease onset^24,44,45^. In contrast, activating nuclear import or inhibiting arginine methylation may reverse these events and delay or prevent the onset of ALS.

## Methods

### Protein expression and purification

GST-Importins were overexpressed in BL21 (DE3) *E. coli* cells, induced with 0.5 mM isopropyl-β-d-1-thiogalactoside (IPTG) for 12 h at 25 °C. Cells were harvested by centrifugation, resuspended in lysis buffer (50 mM Tris pH 7.4, 150 mM NaCl, 1 mM EDTA, 2 mM DTT, 15% glycerol and protease inhibitors) and then lysed with the EmulsiFlex-C5 cell homogenizer (Avestin, Ottawa, Canada). GST-Importins were purified by affinity chromatography using Glutathione Sepharose 4B (GSH; GE Healthcare). GST-Importins were eluted with buffer containing 30 mM glutathione to be used in pull-down assays; these proteins were further purified by anion exchange chromatography followed by size-exclusion chromatography. To purify untagged Kapβ2 and Impβ for ITC experiments, crystallography and turbidity assays, the GST tag of GST-importins was removed by adding Tev protease to GST-importins on the GSH column. The importin proteins are released from the GSH beads and further purified by anion exchange chromatography followed by size-exclusion chromatography (Superdex200, GE Healthcare).

MBP-FUS constructs were expressed in BL21 (DE3) *E. coli* cells (induced with 0.5 mM isopropyl-β-d-1-thiogalactoside (IPTG) for 16 h at 18 °C). Cells were lysed in 50 mM HEPES pH 7.4, 1.5 M NaCl, 10% v/v glycerol, 2 mM DTT (high salt to disrupt association with nucleic acid). MBP-FUS proteins were purified by affinity chromatography using amylose resin (New England BioLabs, Ipswich, MA), eluted with buffer containing 20 mM HEPES pH 7.4, 150 mM NaCl, 2 mM DTT, 10% glycerol, and 20 mM maltose. MBP-FUS constructs were further purified by size-exclusion chromatography (Superdex200, GE Healthcare).

His-tagged Ran (Gsp1 (1–179, Q71L)) was expressed in E. coli BL21 (DE3) cells (induced with 0.5 mM IPTG for 12 h at 20 °C). Harvested cells were lysed with the EmulsiFlex-C5 cell homogenizer (Avestin, Ottawa, Canada) and the protein purified by affinity chromatography on Ni–NTA column. Eluted proteins were loaded with GTP, and further purified by cation exchange chromatography^31,46^.

### Crystallization and structure determination of Kapβ2-FUS(P525L)^PY-NLS^ complex

To assemble the Kapβ2-FUS(P525L)^PY-NLS^ complex for crystallization, bacteria expressing GST-Kapβ2ΔLoop (residues 337–367 were replaced with a GGSGGSG linker) and MBP-FUS(475–526, P525L) were mixed and lysed together in a buffer containing 20 mM Tris pH7.4, 150 mM NaCl, 2 mM DTT, 5 mM EDTA, 2 mM PMSF and 10% glycerol. The complex was purified by affinity chromatography using GSH sepharose beads (GE Healthcare) and amylose beads (New England Biolabs), GST and MBP removed with TEV protease, and the Kapβ2-FUS(P525L)^PY-NLS^ complex purified by gel filtration chromatography using Superdex 200 HiLoad 16/60 (GE healthcare) in a buffer containing 20 mM HEPES pH 7.4, 110 mM potassium acetate, 2 mM magnesium acetate and 2 mM DTT with 20% glycerol. The Kapβ2-FUS(P525L)^PY-NLS^ complex was concentrated to 10 mg/mL for crystallization. Kapβ2-FUS (P525L)^PY-NLS^ crystals were obtained by hanging drop vapor diffusion at 20ºC (1.0 μL protein + 1.0 μL reservoir solution) with a reservoir solution of 1 M Succinic acid (pH7.0), 1%(w/v) PEG MME2000. Crystals were cryo-protected by addition of ~ 25% glycerol, and flash-cooled by immersion in liquid nitrogen. X-ray diffraction data were collected at the Advance Photon Source 19ID beamline in the Structural Biology Center at Argonne National Laboratory using a wavelength of 0.9795 Å. Diffraction data were indexed, integrated, and scaled using XDS^47^. The structure was determined by molecular replacement using PHASER with a search model of human Kapβ2 (chain A from PDB ID 4FDD)^48^. Several rounds of refinement using phenix refine and manual model building with Coot were performed^49,50^. During the refinement process, the X-ray/stereochemistry weight was optimized and TLS refinement was performed. The final model of the Kapβ2-FUS(P525L)^PY-NLS^ complex was validated using Molprobity^51^. Illustrations were prepared with PyMOL (https://pymol.org/2/). Simulated annealing composite omit maps were generated by map tool in PHENIX.

### Turbidity assay

Prior to adding Tev protease, MBP-FUS, ± Importinβs, ± RanGTP, ± Importinα IBB(residues 1–74), ± M9M peptide were mixed in buffer containing 20 mM HEPES pH 7.4, 150 mM NaCl, 2 mM magnesium acetate, 20 μM zinc acetate and 2 mM DTT with 10% glycerol. Tev protease was added to the premixture, to a final concentration of 25 μg/mL then incubated at room temparature for 60 min. Absorbance at 395 nm (OD395 nm) was measured using plate reader. For the Kapβ2 titration turbidity assay, Tev digestion for 8 uM MBP-FUS was initiated in the absence or presence of 2–16 uM Kapb2 at 30 °C. After 1 h, the samples cooled down to 10 °C then measured OD395 nm. All experiments were performed three or four technical repeats and represented as mean ± SD.

### Pull-down binding assays

Pull-down assays were performed by immobilizing 4–8 μM of GST-importins on 50 μL of GSH beads. 4–8 μM of MBP-FUS protein and GSP1 were added the immobilized GST-importins in a total volume of 100 μL containing binding assay buffer (BA buffer; 20 mM HEPES pH 7.4, 110 mM potassium acetate, 2 mM magnesium acetate, 2 mM DTT and 15% glycerol). The protein mix sat for 30 min at 25 °C followed by three washes of a total of 500μL of BA buffer. Proteins bound on the beads were eluted by boiling in SDS sample buffer and visualized by Coomassie stained SDS-PAGE gels. Positive control experiments of GST-importins binding to RanGTP and a negative control experiment of MBP-FUS(371–500) binding to GST immobilized on beads were performed as described above.

### Binding affinities measurement by isothermal titration calorimetry (ITC)

Kapβ2 and MBP-FUS proteins purified as described above. Kapβ2 and MBP-FUS proteins were dialyzed into ITC buffer containing 20 mM Tris–HCl, 150 mM NaCl, 10% Glycerol, 2 mM 2-mercaptoethanol (BME). ITC experiments were performed in an iTC-200 calorimeter (Microcal, LLC, Northampton, MA, USA) with the stirred reaction cell of 202.9 μL held at 20 °C; the first injection was 0.5 μL, followed by twenty 1.9 μL injections. The stirring rate was 750 rpm. Kapβ2 was mostly used at 10 μM in the ITC cell, except for experiments with MBP-FUS(1–500) and MBP-FUS(1–370) where 100 μM and 200 μM Kapβ2 were used, respectively. When 10 μM Kapβ2 was in the ITC cell, 100 μM or 200 μM MBP-FUS proteins were used in the syringe. For experiments with MBP-FUS(1–500) and MBP-FUS(1–370), 10 μM of the MBP-FUS proteins were placed in the ITC cell, and 100 μM and 200 μM Kapβ2 were placed in the syringe, respectively. All ITC experiments were carried out in either duplicates or triplicates. Data were integrated and baseline corrected using NITPIC^52^. The integrated data were globally analyzed in SEDPHAT^53^ using a model considering a single class of binding sites. Thermogram and binding isotherm figures were then plotted in GUSSI^54^.

### Analysis of Flag-tagged FUS fusion proteins localization in cells

All FLAG-tagged FUS constructs used for transient transfections were cloned into the 5′-FLAG mammalian expression vector as previously described^55^. For immunofluorescence microscopy analysis, HeLa cells were seeded onto round 12 mm diameter coverslips in a 24-well plate and transfected with plasmids using Lipofectamine 3000 (Thermo Fisher Scientific), according to the manufacturer’s instructions. 20 μM AdOx (Sigma-Aldrich) was added to cells 16 h prior to transfection. The cells were usually fixed 24 h after transfection with 4% paraformaldehyde prepared in PBS. The fixed cells were permeabilized with 0.5% Triton X-100 prepared in PBS, rinsed, and blocked with 1% BSA prepared in PBS containing 0.1% Tween-20 (PBST). The slides were incubated at 4 °C overnight with FLAG antibody (Sigma-Aldrich). Unbound antibodies were removed by washes with PBST and the slides were then incubated with Alexa Fluor 488 secondary antibody (Thermo Fisher Scientific), washed, and mounted with Fluoro-KEEPER Antifade Reagent (Nacali tesque). Immunostained cells were examined using an inverted fluorescence microscope (EclipseTi2; Nikon) equipped with a 40 × 0.6 NA air objective. Z-stack images were obtained in GFP and DAPI channels using a step size of 0.6 µm (total 6 µm). Nuclear region was defined by DAPI and sum of fluorescence intensities from FUS were analyzed using General Analysis tool in NIS-Element software (Nikon). 2D deconvolution tool in NIS-Element software was only applied for representing images in figure. Images and quantification shown are from one experiment, but the results were reproduceable. Statistical analysis was carried out using the t-test with SigmaPlot 14 software.

### Analysis of EYFP_2_-tagged FUS fusion proteins localization in cells

Expression constructs: All EYFP_2_ constructs used for transient transfections were cloned into the pEYFP_2_ mammalian expression vector as previously described in Fu et al. ^56^. The Bimax2 peptide inhibitor and PRMT1 were was cloned into pTagRFP-C (Evrogen), and cloning of the M9M peptide inhibitor was as described in Cansizoglu et al.^27^.

Cell culture, transfection and inhibitor treatment: HeLa cells from the American Type Culture Collection were cultured in DMEM (Sigma-Aldrich) supplemented with 10% fetal bovine serum (FBS; Sigma-Aldrich) and 1% antibiotic–antimycotic (Life Technologies, Thermo Fisher Scientific) at 37 °C in 5% CO2. Cells were plated at roughly 70% confluency cells per well into glass-bottom 24-well culture plates (Phenix Research Products). Transfections were performed according to the manufacturer’s instructions (Lipofectamine 3000, Life Technologies, Thermo Fisher Scientific). Co-transfection with RFP-Bimax2, MBP-M9M or RFP-PRMT1 was conducted using a transfection mixture of plasmid DNAs at ratio of 1:1. For all transient transfections, cells were analyzed 18 h post‐transfection. AdOx (Sigma) and Importazole (IPZ; Sigma) were dissolved in DMSO and was added to cells upon plating and 4 h post transfection at a concentration of 20 μM. Importazole (Sigma) was used at 20 μM for 18 h post-transfection. 0.4% (v/v) DMSO was used in control experiments.

#### Immunofluorescence, confocal image acquisition, image quantification and statistics

Cells were fixed with 4% formaldehyde and permeabilized with 0.1 Triton X-100 1 prior to fluorescence microscopy. The primary antibody MBP monoclonal antibody (Cell Signaling Technology, 1:1000 and the secondary Alexa Fluor 594‐conjugated anti‐mouse antibody (Cell Signaling Technology, 1:1000) were used with Hoechst 33,342 (Life Technologies, Thermo Fisher Scientific) for nuclear counterstaining. Confocal images of live and fixed HeLa cells were obtained using a spinning disk confocal microscope system (Nikon-Andor) with a 40 × 0.6 NA air objective and the MetaMorph software. Z-stack images were obtained in the YFP, RFP and Hoechst channels using a step size of 0.6 μm (total z size 18 μm). In addition, a single differential interference contrast (DIC) image was taken in the middle of the z-stack. Nuclear and cytosolic localization analysis and quantification were performed by custom macro developed with ImageJ (v1.53C, NIH) described in Fu et al.^56^. If necessary for printing, brightness and contrast were linearly enhanced using Adobe Photoshop's Level tool. Images and quantification shown were from one experiment, but are representative of three independent experiments. Statistical analysis was carried out using the one‐way ANOVA with a Tukey post test (Prim8, GrapPad). Images and quantification shown are from one experiment, but are representative of three independent experiments.

## Supplementary Information

Supplementary Information.
