# Supplementary material for: Mechanism of karyopherin-β2 binding and nuclear import of ALS variants FUS(P525L) and FUS(R495X)
Source: Sci Rep. 2021 Feb 12;11:3754. doi: 10.1038/s41598-021-83196-y (PMC7881136; doi:10.1038/s41598-021-83196-y)
Supplement: Supplementary file 1 — Supplementary Information. [file 41598_2021_83196_MOESM1_ESM.pdf]

Supplementary Information

Mechanism of Karyopherin-β2 binding and nuclear import of ALS variants FUS(P525L) and FUS(R495X)

Abner Gonzalez[1], Taro Mannen[2], Tolga Çağatay[1], Ayano Fujiwara[2], Hiroyoshi Matsumura[2], Ashley B. Niesman[1], Chad A. Brautigam[3], Yuh Min Chook[1,*] and Takuya Yoshizawa[2,*]

[1]Department of Pharmacology, University of Texas Southwestern Medical Center, Dallas, TX, USA.

[2]College of Life Sciences, Ritsumeikan University, Shiga, JAPAN

[3]Department of Biophysics, University of Texas Southwestern Medical Center, Dallas, TX, USA.

*Co-corresponding authors,

t-yosh@fc.ritsumei.ac.jp

yuhmin.chook@utsouthwestern.edu

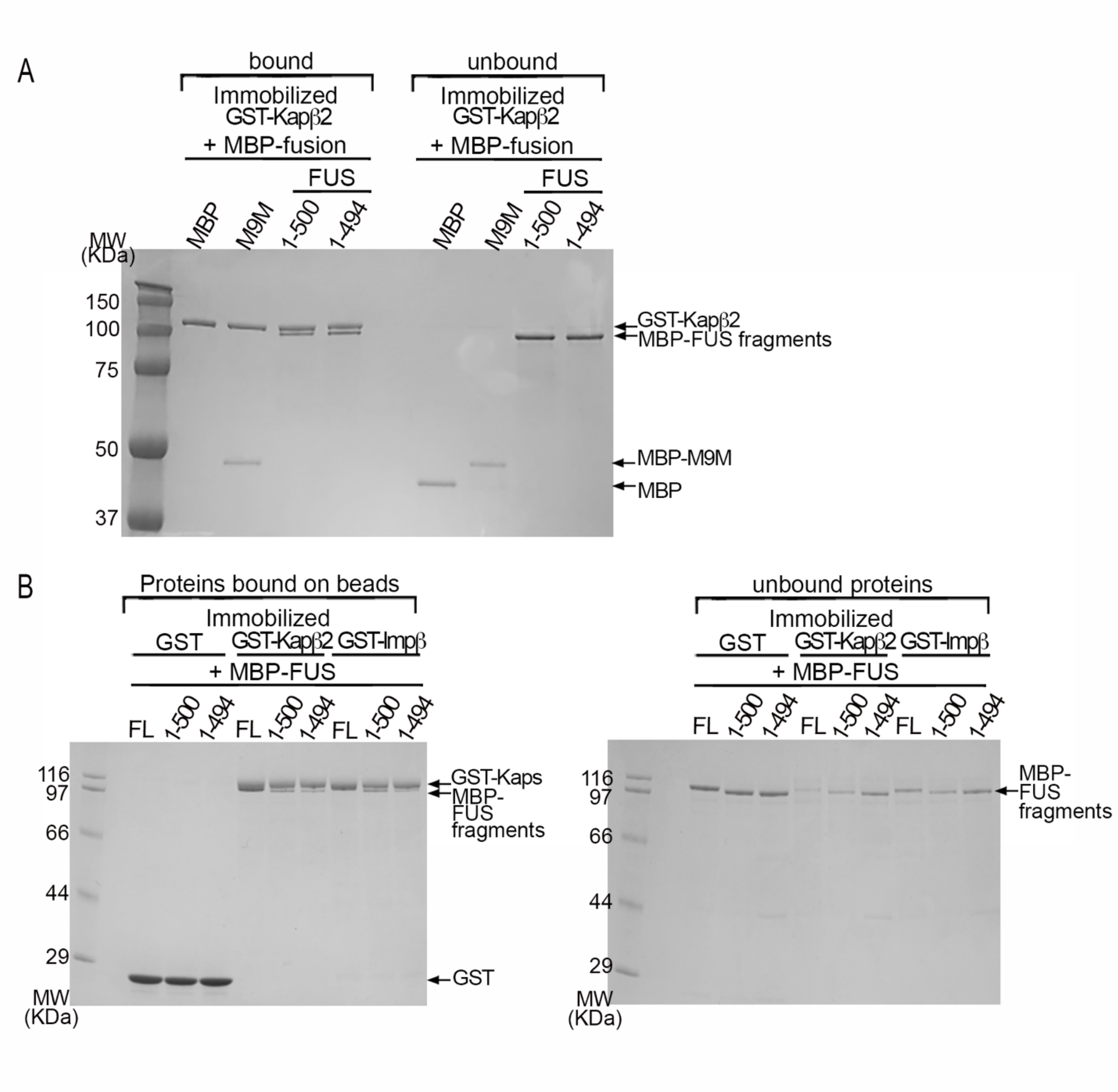

Supplementary Figure 1. Pull-down binding assays of FL FUS, FUS(1-500) and FUS(R495X) or FUS(1-494) binding to importins. A) Image of the entire gel showing the pull-down binding assay of MBP-FUS R495X and MBP-FUS 1-500 to immobilized GST-Kapβ2 (left; a portion of this gel is shown as the middle panel of Figure 2B) and a gel showing 4μl of each unbound fraction in the experiment (right).**B)** Image of the entire gel (a portion of this gel is shown as the right panel of Figure 2B) and a gel of the unbound proteins (10 μL of each unbound fraction).

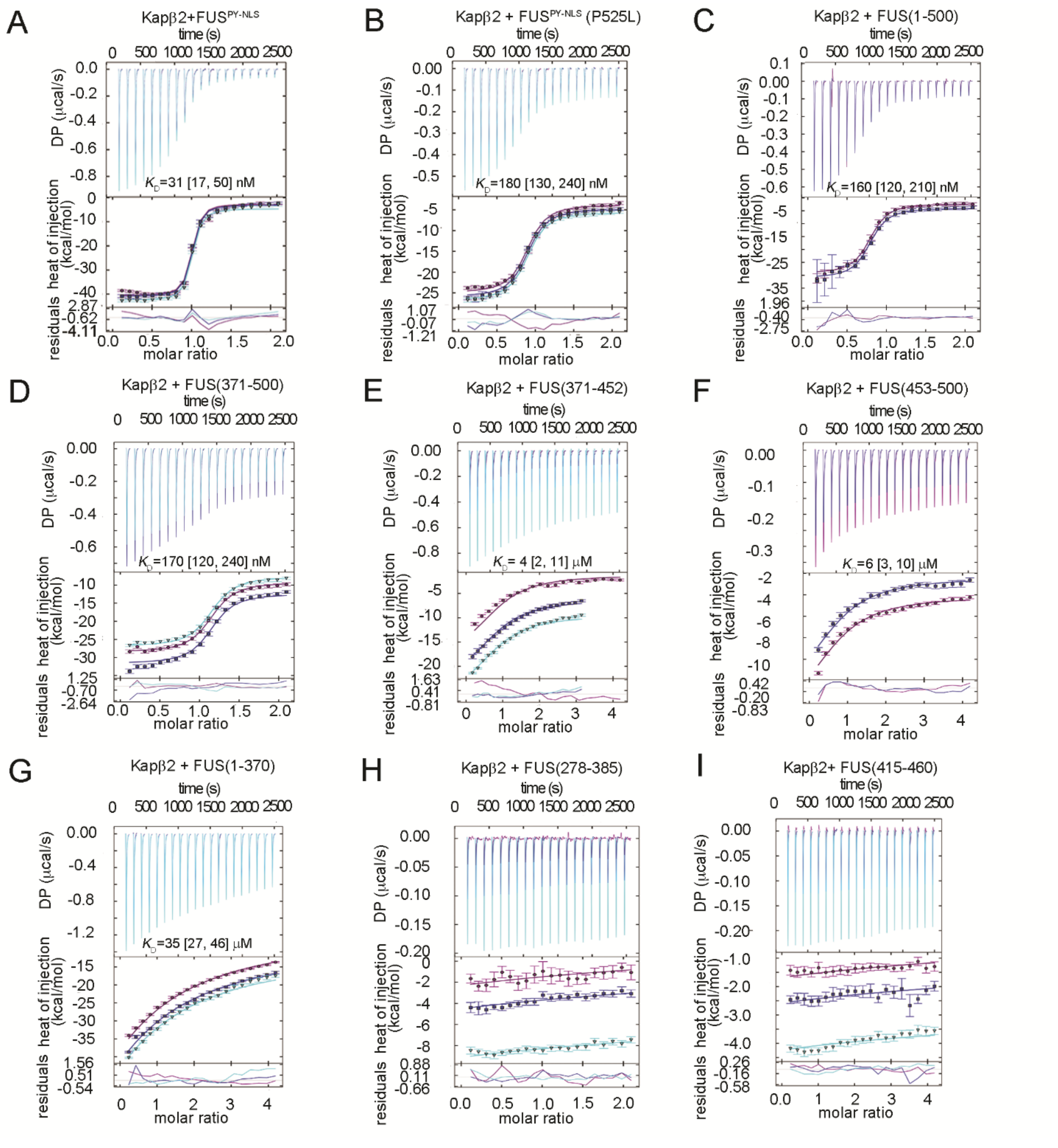

Supplementary Figure 2. ITC analysis of Kapβ2 binding affinity for MBP-FUS proteins. A-I) GUSSI output image for the global analysis of Kapβ2 binding to MBP-FUS constructs. Top panel illustrate the reconstructed thermogram from NITPIC, middle panel shows the binding isotherm and bottom panel shows the residuals. All experiments were performed in duplicate or triplicate on ITC200.

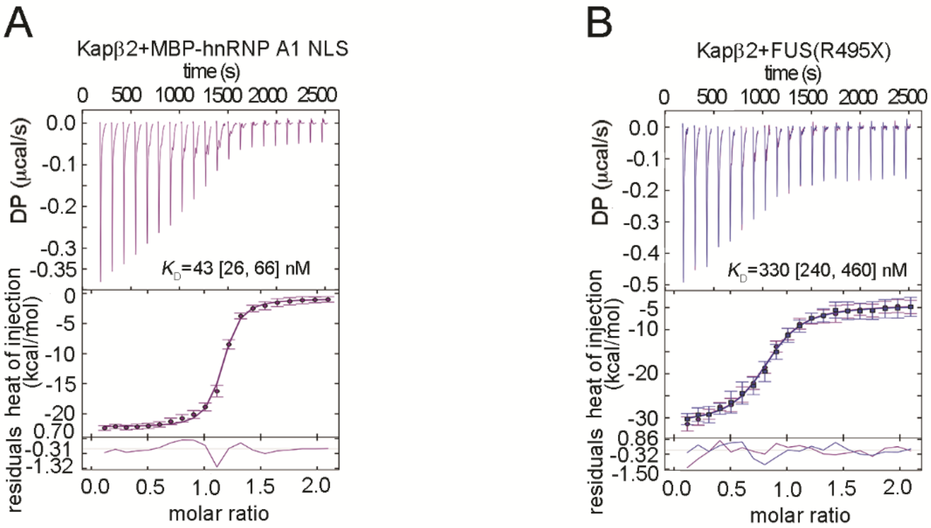

Supplementary Figure 3. ITC analysis of Kapβ2 binding affinity for MBP-FUS(R495X). GUSSI output image for the global analysis of Kapβ2 binding to MBP-hnRNP A1 NLS (**A**) and to MBP-FUS(R495X) (**B**), which show the reconstructed thermograms from NITPIC (top panels), the binding isotherms (middle panels) and the residuals (bottom panels). The ITC experiments were performed using a MicroCal ITC-200 calorimeter. Proteins were dialyzed before experiment into ITC buffer containing 20 mM Tris-HCl, 150 mM NaCl, 10% Glycerol, 2 mM 2-mercaptoethanol. The experiments were performed with **A**) Kapβ2 (202.9 mL of 10 μM protein) and **B**) MBP-FUS(R495X) (202.9 mL of 10 μM protein) in the ITC cell at 20 °C. The first MBP-hnRNP A1 injection (**A**) and Kapβ2 injection (**B**) were 0.5 μL followed by 20 injections of 1.9 μL each, stirring rate 750 rpm. The experiment for **B** was carried out in duplicate.

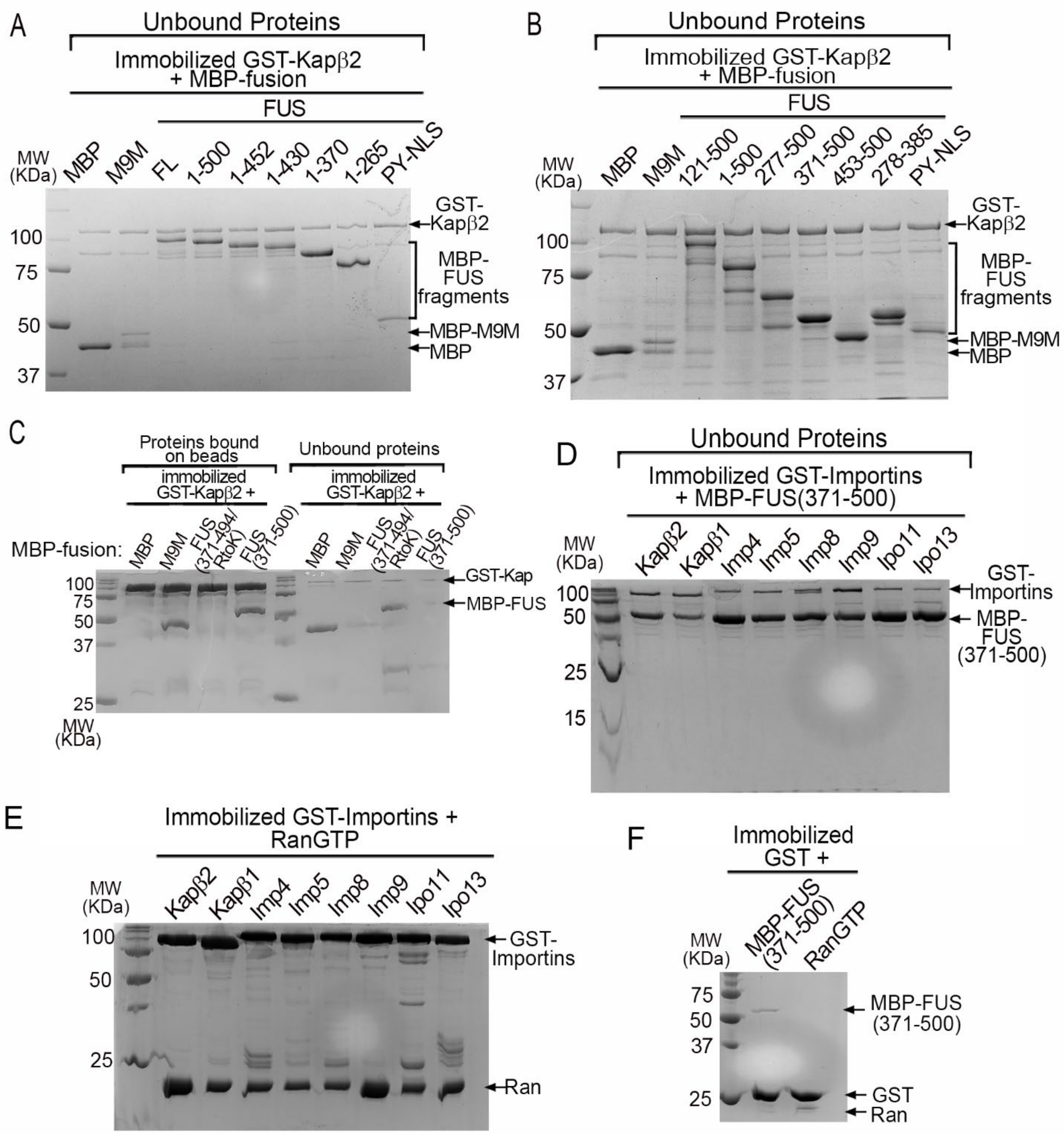

Supplementary Figure 4. Pull-down binding assays of MBP-FUS proteins binding to importins. A, B) Unbound proteins from each of the pull-down binding experiments in Figure **4B, C** (6 μL from each unbound fraction; immobilized GST-Kapβ2 binding to various to MBP-FUS fragments). **C)** Pull-down binding assay of MBP-FUS(371-494 /RtoK) and MBP-FUS(371-500) to immobilized GST-Kapβ2. 8 μL of each unbound protein fractions loaded onto SDS-PAGE gel. **D)** Unbound proteins from the pull-down binding experiments in Figure **4E** (MBP-FUS(371-500) binding to various immobilized GST-importins; 8 μL of each unbound protein fractions). **E)** Control experiments (pull-down binding assay) of RanGTP binding to all the immobilized GST-importins used in Figure **4E**. **F)** Control experiment (pull-down binding assay) of immobilized GST binding to MBP-FUS(371-500) and to RanGTP. All proteins are visualized by Coomasie-stained SDS-PAGE.

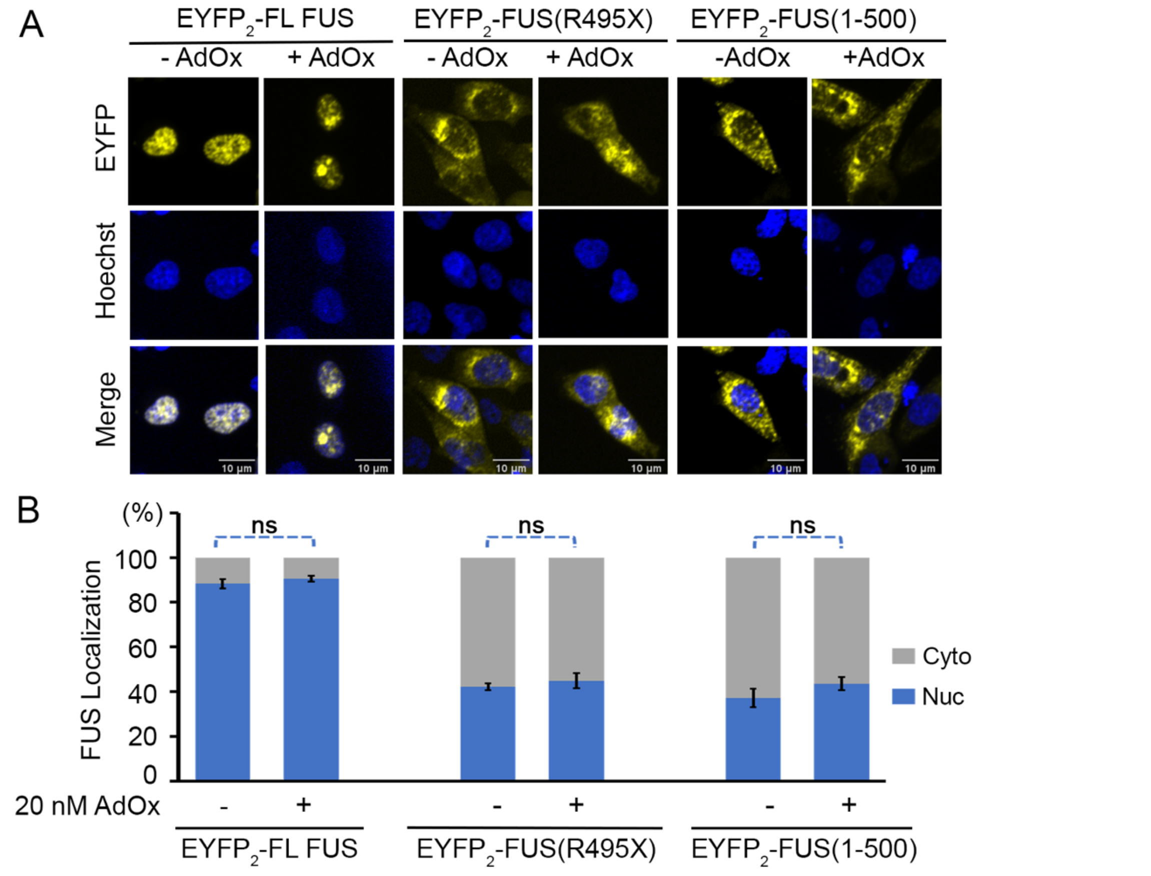

Supplementary Figure 5. Localization of full-length FUS, FUS(R495X) and FUS(1-500) in HeLa cells. A) Confocal microscopic images of live HeLa cells expressing EYFP2-FL FUS or EYFP2-FUS(R495X) or EYFP2-FUS(1-500), with or without methylation inhibitor AdOx (20 μm) treatment. Hoechst 33342 was used as nuclear counter stain. Scale bar = 10 μm. **B)** Bar diagram of relative percentage of nuclear (Nuc) and cytoplasmic (Cyto) fluorescence intensity in cells is shown with the mean ± SEM, n = 10-14. Ordinary one-way ANOVA test was performed for statistical analysis using GraphPad software. n.s, not significant.

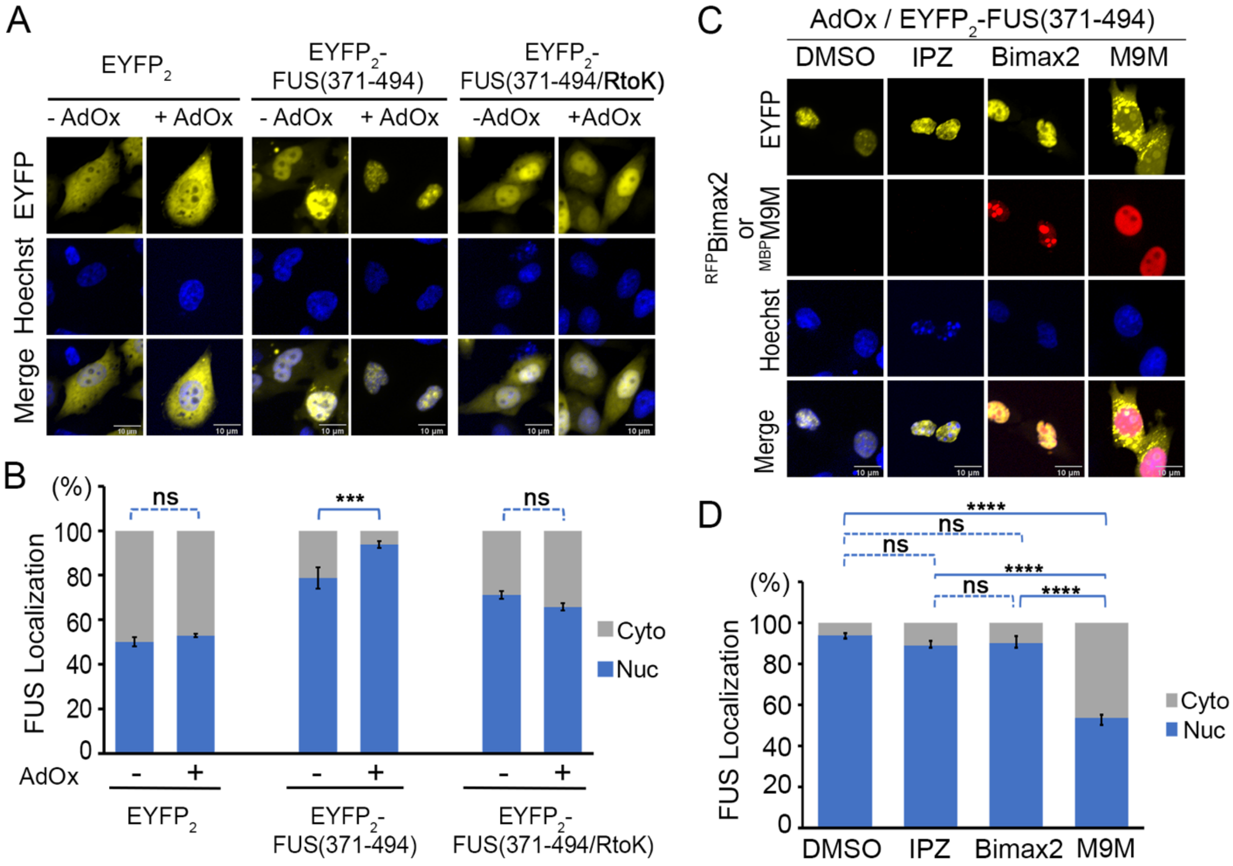

Supplementary Figure 6. Localization of FUS(371-494) in HeLa cells. A) Confocal microscopic images of live HeLa cells expressing $EYFP_2$ or $EYFP_2$-FUS(371-494) or $EYFP_2$-FUS(371-494/RtoK) with or without methylation inhibitor AdOx (20 µm) treatment. Hoechst 33342 was used as nuclear counter stain. Scale bar = 10 µm. **B)** Bar diagram of relative percentage of nuclear (Nuc) and cytoplasmic (Cyto) fluorescence intensity in cells is shown with the mean ± SEM, n = 10-14. **C)** Localization of $EYFP_2$-FUS(317-494) in the presence of importin β1 inhibitor (Importazole), importin α inhibitor (expressed Bimax2 peptide) and Kapβ2 inhibitor (expressed M9M peptide). The HeLa cells were first treated with AdOx. Expression of the peptide inhibitors were either monitored directly (RFP-BiMax2, second row) or by direct immunofluorescence (MBP-M9M via Alexa 565 second antibody, second row).Scale bar = 10µm. **D)** Bar diagram of relative percentage of nuclear (Nuc) and cytoplasmic (Cyto) fluorescence intensity in cells is shown with the mean ± SEM, n = 10-14. Ordinary one-way ANOVA test was performed for statistical analysis using GraphPad software. Significant differences compared with the corresponding control samples are indicated ****p < 0.001. ns, not significant.

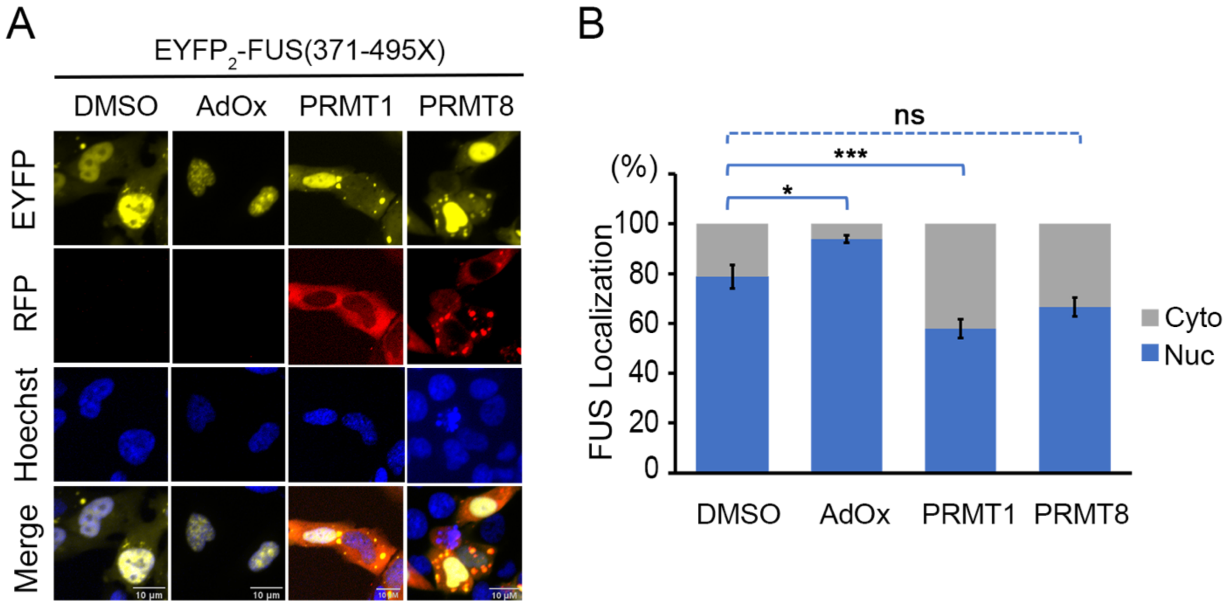

Supplementary Figure 7. Localization of FUS(371-494) and overexpression of arginine methyltransferases in HeLa cells. A) Localization of EYFP2-FUS(317-494) in live HeLa cells treated with methylation inhibitor AdOx (20 μm) (column 2), or in cells co-expressing either RFP-PRMT1 (column 3) or RFP-PRMT8 (Column 4). Hoechst 33342 was used as nuclear counter stain. Scale bar = 10 μm. **B)** Bar diagram of relative percentage of nuclear (Nuc) and cytoplasmic (Cyto) fluorescence intensity in cells is shown with the mean ± SEM, n = 10-14. Ordinary one-way ANOVA test was performed for statistical analysis using GraphPad software. Significant differences compared with the corresponding control samples are indicated ***p < 0.001. ns, not significant.

Supplementary Table 1. Crystallographic statistics for the Kapβ2-FUS(P525L)$^{PY\text{-}NLS}$ complex

Space group	$P2_12_12$
Cell dimensions:<br>a, b, c (Å)<br>α, β, γ (°)	 129.2, 158.4, 68.7<br>90, 90, 90
**Data collection**	
Wavelength (Å)	0.97915
Resolution range (Å)	47.06 - 2.70 (2.80 - 2.70) [a]
Completeness (%)	99.69 (99.08)
Redundancy	7.0 (5.8)
R-merge (%)	6.2 (86.4)
R-pim (%)	2.6 (38.0)
I/σ(I)	19.3 (2.2)
CC1/2 (%)	99.9 (81.5)
**Refinement**	
Resolution range (Å)	47.06 - 2.70 (2.80 - 2.70)
No. of reflections	39,427 (3,860)
R-work (%)	19.7 (29.9)
R-free (%)	22.7 (33.1)
RMDSs:<br>Bond lengths (Å)<br>Bond angles (°)	 0.01<br>1.46
Average overall B factor (Å^2)	90.23
Ramachandran plot:<br>Favored region (%)<br>Allowed region (%)<br>Outliers (%)	 95.06<br>4.940.00
**Model contents**	
Protomers in ASU	
Kap β 2	1
No. of Kap β 2 residues	836
No. of Kap β 2 atoms	6,656
FUS PY-NLS(P525L)	1
No. of FUS PY-NLS(P525L)	20
No. of FUS PY-NLS(P525L) atoms	171
No. of water atoms	0
**PDB Accession Code**	7CYL

[a] Values in parentheses correspond to the highest-resolution shell.
